# Effects of *Moringa oleifera* Leaf Extracts on *Xanthomonas campestris* pv. *campestris*

**DOI:** 10.3390/microorganisms9112244

**Published:** 2021-10-28

**Authors:** Riccardo Fontana, Anna Caproni, Raissa Buzzi, Mariaconcetta Sicurella, Mattia Buratto, Francesca Salvatori, Mariangela Pappadà, Stefano Manfredini, Anna Baldisserotto, Peggy Marconi

**Affiliations:** 1 Department of Chemical, Pharmaceutical and Agricultural Sciences, University of Ferrara, 44121 Ferrara, Italy; fntrcr1@unife.it (R.F.); anna.caproni@edu.unife.it (A.C.); mariaconcetta.sicurella@unife.it (M.S.); mattia.buratto@unife.it (M.B.); francesca.salvatori@unife.it (F.S.); mariangela.pappada@unife.it (M.P.); 2 Department of Life Sciences and Biotechnology, University of Ferrara, 44121 Ferrara, Italy; raissa.buzzi@unife.it (R.B.); anna.baldisserotto@unife.it (A.B.)

**Keywords:** *Xanthomonas campestris*, *Moringa oleifera* Lam, antimicrobial activity, nutraceutical, cosmeceutical

## Abstract

*Xanthomonas campestris* pv. *campestris* (Xcc) is a Gram-negative bacterium belonging to the Xanthomonodaceae family, causing black rot in crucifers. To control this pathogen, the study investigated the effect of different leaves extracts of *Moringa oleifera* Lam., a tropical plant, well known for its food properties and with countless applications in many different fields, from nutraceutical (hypoglycemic) to the cosmetic (sunscreen) properties. Nevertheless, several studies pointed to its antibacterial action against both Gram-negative and Gram-positive bacteria. Many bioactive compounds, including flavonoids, phenolic acids, alkaloids, isothiocyanates, tannins and saponins, contained in these extracts, are responsible for its countless activities. The analyses carried out in this study show that the methanolic, hydroalcoholic and hydroalcoholic maltodextrin extracts have both bacteriostatic and bactericidal effects at concentrations of 0.5, 0.5 and 0.1 mg/mL respectively. In particular, the study shows how all extracts can alter membrane permeability, to adversely affect swarming motility, and to alter biofilm formation in Xcc. The in planta experiments showed a reduction of the necrosis area in the infected radishes, although the ability of the extracts to be absorbed by root systems is yet to be understood, in order to reach the target point.

## 1. Introduction

*Xanthomonas campestris* pv. *campestris* (Xcc) is a Gram-negative bacterium belonging to Xanthomonodaceae family, causing black rot in crucifers [1]. Once it reaches the target site in the plant, it damages plant cells causing maceration of the tissues and obstruction of the xylematic vessels [2]. This disease is one of the most widespread and destructive in the world, with an economic impact in many geographical areas, comprising Brazil, Ethiopia, South Africa, Belgium, Germany, Sweden, France, Netherlands, Italy, United States, United Kingdom, Nepal, China, Taiwan, Canada, Australia, and India [3]. Xcc infection is particularly harmful and difficult to control due to the numerous virulence factors that characterize the bacterium that, specifically, has a single polar flagellum that allows the bacterium, once inoculated, and penetrated the plant, to move and reach the vascular system [4]. Xcc has developed a unique quorum sensing system, which plays a key role in regulating the biosynthesis of bacterial virulence factors [5]. Once the target site is reached, colonization takes place by the action of pili and the production of extracellular polysaccharides, including xanthan gum, and extracellular enzymes, such as cellulase, protease, polygalacturonase, amylase, hemolysin and hemagglutinin [6]. In addition, among the systems that substantially participate in the pathogenicity mechanisms, the secretion system type III (SST3) (also known as secretion system Hrp) should be also taken into account, it interferes with plant defense mechanisms [7].

Numerous investigations have suggested that *M. oleifera* Lam. exerts an antibacterial action against both Gram-negative and Gram-positive bacteria. [8,9] Many bioactive compounds, including flavonoids, phenolic acids, alkaloids, isothiocyanates, tannins and saponins, are responsible for its antimicrobial activity [9]. All the compounds found in *M. oleifera* Lam. extracts (MOEs) participate in the countless properties that the plant can offer. In particular, high concentrations of flavonols such as quercetin, kaempferol and rutin and flavones, such as apigenin are observed. [10,11] Several glycosylated flavonoids have also been identified, including quercetin-3-O-glucoside, kaempferol-3-O-glucoside and kaempferol-3-O-rutinoside [12]. In the dried leaves has been found the presence of a high content of phenolic acids such as gallic acid, chlorogenic acid, ellagic acid, ferulic acid and caffeic acid [13]. Among these, chlorogenic acid, acts by interfering with the processes of gluconeogenesis and glycogenolysis and reducing cholesterol and triglycerides [14]. Various alkaloids were found in *M. oleifera* Lam. leaves including marumoside A, marumoside B, α-L-ramnopiraonsyl-vincosamide, phenylacetonitrile and its glucopyranosilic derivative [15]. The dried leaves are an excellent source of carotenoids and retinol, precursors of vitamin A, vitamins B, vitamin E, and vitamin C [14]. As for the antimicrobial activity, some studies highlighted this property for different MOEs, i.e., in 2016, Elgamily et al. showed how different extracts from seeds, leaves and roots were able to inhibit *Staphylococcus aureus* and *Streptococcus mutans* growth [16].

Given the economic impact that the disease causes in many regions, combined to the research of alternative treatments to the use of chemicals or antibiotics, we have an ongoing research program (SUSTANIA) aimed to investigate plant extracts selected on the base of their sustainable source and safe by design (renewable plant parts, not at risk of extinction, high productivity and possibly used for food or cosmetics). In this context, and based on previous research [17], we have decided to investigate the effect of different leaves extracts of *Moringa oleifera* Lam. to alter membrane permeability, to affect swarming motility, and to alter biofilm formation in Xcc.

## 2. Materials and Methods

### 2.1. Plant Material and Extraction Methods

*M. oleifera* Lam. leaves were harvested in August 2019 (Lot number: 19E0854X1809). After collecting, the leaves were dried by Evra S.r.l. (Loc. Galdo, 85044 Lauria, Italy). Dried sample was packaged and sent to our laboratory. Upon arrival, dried leaves were ground to a fine powder with a mortar and stored at −80 °C. The extraction processes were carried adapting previously described methods [18,19]. The extracts were stored at −18 °C until use. The water and the hydroalcoholic extracts with maltodextrin (WMD-MOE and HAMD-MOE respectively) were obtained by Evra S.r.l. company.

- Hydroalcoholic extract (HA-MOE): about 10 g of powder were mixed with 200 mL of hydroalcoholic solution (ethanol:water, 70:30) at room temperature for 1 h under magnetic stirring. The residue was filtered and concentrated in vacuum to provide the desired hydroalcoholic dry extract.
- Methanolic extract (MeOH-MOE): about 5 g of powder were mixed with 100 mL of methanol and subjected to two sonication cycles (40 °C, 60 min, 80%) with subsequent centrifugation. The supernatant was concentrated under vacuum to obtain the desired product.
- Decoction (In-MOE): 10 g of powder are left to infuse for 30 min with 150 mL of deionized water, previously brought to a boil. The decoction solution was filtered and lyophilized to obtain the dried water extracts.
- Water extract with maltodextrins (WMD-MOE): dried M. oleifera Lam. leaves were extracted with water (raw material:solvent 1:10) for 45 min at 65 °C. After filtration, concentration and pasteurization, the extract was spray dried using maltodextrin, obtaining a fine powder.
- Hydroalcoholic extract with maltodextrins (HAMD-MOE): dried M. oleifera Lam. leaves were extracted with 50% ethanol (raw material:solvent 1:10) for 45 min at 45 °C. After filtration, concentration and pasteurization, the extract was spray dried using maltodextrin, obtaining a fine powder.

### 2.2. Total Polyphenol Content

To evaluate the polyphenol content of the extracts was evaluated by the adapted and optimized procedure of Folin–Ciocalteu [20]. Gallic acid was used as a standard to obtain a calibration curve (0–500 ppm). 20 µL of each extract were mixed with Folin-Ciocalteu reagent diluted in water (1.5 mL) and incubated at room temperature for 5 min. Then 300 µL of a sodium carbonate solution was added and then incubated at room temperature in the dark for a further 90 min. Finally, the absorbance is measured by UV-VIS spectrophotometer at 765 nm (UV-31 Scan ONDA) against a blank containing distilled water instead of the extracts. The results were expressed as equivalent to micrograms of gallic acid equivalent (GAE) per milligram of a sample (µg of GAE/mg of dry extract).

### 2.3. Determination of Total Flavonoid Content

Total flavonoid content was determined following the method by Singh et al. (2012) and Jinting et al. (2017) [21,22]. Accurately, 1 mL of sample or standard was diluted with 4 mL distilled water and 0.3 mL of 5% sodium nitrate solution was added. After 6 min, 0.3 mL of a 10% aluminum chloride solution was added to the mixture. The mixture was incubated at room temperature for 5 min. Then, 2 mL of 1 M sodium hydroxide was added to the mixture after 5 min of incubation. The mixture was vortexed thoroughly and the absorbance of the yellow color was measured at 510 nm against a blank by using UV-31 Scan ONDA Spectrophotometer. Quercetin was used for the calibration curve with a concentration range of 10–1000 μg/mL. Results were expressed as micrograms quercetin equivalent (μg QE g-1) of dried extract. All experiments were carried out in four replicates.

### 2.4. Characterization of Polyphenols

#### 2.4.1. Preparation of Standard Solutions and Sample

Standard solutions of chlorogenic acid, rutin, ellagic acid, ferulic acid and quercetin were prepared and diluted in methanol to obtain the final concentration in the range 0.625–80 µg/mL. A carefully weighed aliquot (20 mg) of each extract was dissolved in methanol or methanol-water. Each solution was filtered through a 0.45 nylon membrane filter and subsequently analyzed in triplicate by HPLC.

#### 2.4.2. HPLC Apparatus and Chromatographic Conditions

HPLC analysis was performed using an Agilent 1100 Series HPLC System equipped with a G1315A DAD and with a Hydro RP18 Sinergi 80A column (4.6 × 250 mm, 4 µm) from Phenomenex. Separation was monitored with absorbance detection at a wavelength of 254 ± 8 nm. The elution was performed on a gradient solvent using solvent A (water 0.01 M H_3_PO_4_) and solvent B (acetonitrile 0.01 M H_3_PO_4_). The ratios were as follows: 90:10 (A/B) to 80:20 (A/B) in 5 min, held for 5 min, 80:20 (A/B) to 20:80 (A/B) in 10 min, 20:80 (A/B) to 90:10 (A/B) in 2 min. The flow rate was 1.2 mL/min at room temperature. The injection volume for all samples and standards was 5 µL. The quantitative HPLC analysis was calculated, for each compound, according to its peak area.

### 2.5. X. campestris pv. campestris Strains and Culture Conditions

Xcc isolates used in this study were donated from the Emilia-Romagna Phytosanitary Agency and strain 3586 was used as a control. The strains were isolated from Brassica seeds from Bologna, Ravenna and Forlì-Cesena provinces, as specified in Table 1. Stocks of the bacterial strains were conserved at −80 °C in Luria-Bertani (LB) broth with 50% glycerol. During the study, bacteria were plated on R2A Agar (Scharlab Italia, Riozzo di Cerro al Lambro, MI, Italy, 18.1 g/L) or LB agar (Liofilchem, Roseto degli Abruzzi, TE, Italy, 30 g/L) and incubated at 25 °C/28 °C.

### 2.6. Determination of the Minimum Inhibitory Concentration (MIC) of Different M. oleifera Lam. Extracts

The MIC is defined as the lowest concentration of a chemical molecule that blocks visible bacterial growth after an overnight incubation. In order to determine the MIC, the microdilution method was used, as described by Akhlaghi et al. [23]. Xcc was cultured in LB broth overnight at 25 °C, 160 rpm. 80 µL of MOEs (5 mg/mL stock concentration), were added to 120 µL of LB broth, in order to obtain 2 mg/mL concentration of MOEs in the first well of each row in 96-well plates (Costar Corning, Corning, NY, USA). The extracts were diluted in the 96-well microplate to obtain a range of concentrations from 2 mg/mL to 0.001 mg/mL, in a total volume of 200µL. Then, 10µL of the overnight culture were inoculated into each well, standardized being a 10^4^ CFU/mL inoculum. The microplate was then incubated for 48 h at 25 °C. The MIC was determined as the lowest concentration of MOE at which no increase in turbidity occurred. All the analysis were performed from data obtained from three different experiments in triplicate.

### 2.7. Membrane Permeability Assay

Bacterial suspensions were grown overnight in LB broth for 24 h at 25 °C. After incubation, 1 × 10^5^ CFU/mL of bacteria was placed in four different eorfs containing *M. oleifera* Lam. extracts at concentrations corresponding to 2 × MIC, 1 × MIC, 1/2 × MIC and 1/4 × MIC. These suspensions were incubated for 180 min. The procedure was repeated by varying the incubation times, so that the bacterial suspensions, containing the various concentrations of the different extracts, were incubated at for 120 min, 60 min and 5 min respectively. After the incubation time, the suspensions were centrifuged for 5 min at 14,000 rpm, and then wash with PBS 1×. The pellet was then resuspended with propidium iodide (0.5%) and incubated for 15 min avoiding exposure of the suspension to light sources. Then, each suspension has been plated into 96-well plates, and the values were read through a fluorescence microplate reader (Tecan-Fluoroscan, Tecan Italia, Cernusco sul Naviglio, MI, Italy) [24]. All the analysis were performed from data obtained from three different experiments in triplicate.

### 2.8. Swarming Motility Assay

Bacterial suspensions were grown overnight in LB broth for 24 h at 28 °C. After incubation, bacteria were centrifuged and washed three times with PBS 1× (Sigma Aldrich, St. Louis, MO, USA). Then, the pellet of bacteria was resuspended in PBS. Then, bacterial suspensions were diluted in water (1:10 dilution) and 10µL (corresponding to 1 × 10^5^ CFU/mL) of the diluted suspension were plated in the center of a LB soft-agar plate (agar 0.4%), containing the MOEs in non-lethal concentration, as described by Chen et al. [25]. The swarming was then quantified by measuring the diameter of the swarmed zone and measuring the length from the inoculation point to the edge of the swarmed zone, after 48 and 72 h of incubation at 25 °C. All the analysis were performed from data obtained from three different experiments in triplicate.

### 2.9. Biofilm Formation

Effects on biofilm formation were determined by the microplate assay with crystal violet, as described by Wilson et al. [26]. Xcc suspensions, containing 10^6^ CFU/mL, were inoculated in LB broth with MOEs at non-lethal concentration in a 96-well U-bottom microplate for 72 h at 25 °C. After the incubation time, the growth media, MOEs and planktonic cells were removed from the plate and washed with sterile deionized water. Crystal violet 1% was added to each well and incubated for 30 min at room temperature. Then, the dye solution was removed by washing the plate several times with deionized water. 200 µL of decoloring solution (90–95% ethanol) were then added to each well and incubated for 15 min at room temperature to increase crystal violet solubility. The 96-well plate content was then transferred to a new clean microplate and biofilm formation was quantified by reading the absorbance at 570 nm in a microplate reader (Tecan-Sunrise, Tecan Italia, Cernusco sul Naviglio, MI, Italy). All the analysis were performed from data obtained from three different experiments in triplicate.

### 2.10. Effects of MOE on Radishes

Xcc strains were grown overnight in 5 mL of LB broth at 25 °C for 24 h, centrifuged and then resuspended with 1 × PBS in order to reach a bacterial concentration of 5 × 10^7^ CFU/mL. Radishes were superficially sterilized with a 0.7% solution of sodium hypochlorite, delicately perforated with a sterile 10 µL tip and inoculated with 5µL of the bacterial suspension. After the drop had dried, the MIC concentration of each active MOE was inoculated in the same hole. The radishes were then incubated at room temperature (20–22 °C) for 6 days. After the incubation time, the radishes were cut in order to record the necrosis area around the inoculation point. Fiji IMAGEJ was used to quantify the necrosis area [27].

### 2.11. Statistical Analysis

All tests were performed three times in triplicate, and the statistical analysis was performed using one-way ANOVA followed by Dunnett’s multiple comparisons test with GraphPad Prism version 9.0.0 for MacOS, GraphPad Software, San Diego, CA, USA, www.graphpad.com (accessed on 8 January 2021), with *p* ≤ 0.05 to identify significant differences.

## 3. Results

### 3.1. Quantitative and Qualitative Estimations of Polyphenols

For the current study, five different extracts of Moringa oleifera leaves were prepared and analyzed: hydroalcoholic, methanolic, infusion, hydroalcoholic extract with maltodextrins, and water extract with maltodextrins. The analyzes carried out in this study, in particular the Folin–Ciocalteu test and the total flavonoid content test, allowed to highlight a good content of polyphenols in all the samples tested (Table 2). It was therefore decided to carry out a characterization by HPLC which allowed to identify and quantify some of the polyphenols present. In particular, the analyses focused on active ingredients such as chlorogenic acid, ellagic acid, ferulic acid, rutin and quercetin, polyphenols of interest for their antimicrobial activity [19]. The presence of these phenols had already been previously highlighted in *M. oleifera* Lam. leaf extracts from Senegal [17].

Overall (Table 3) the most present active ingredients were found to be ferulic acid, rutin and also chlorogenic acid, except for the WMD-MOE extract that showed the lowest values compared to all the other samples. Specifically, HA-MOE was certainly the one that showed a higher number of polyphenols, presenting an excellent percentage of rutin and ferulic acid. HAMD-MOE and MeOH-MOE follow in terms of percentages of polyphenols: the first showed a good presence of chlorogenic acid, ellagic acid and ferulic acid, while the methanolic extract showed a peak of rutin and ferulic acid. In-MOE and WMD-MOE both reveal the lowest polyphenolic profile of all the samples analyzed, the significant percentages of active ingredients were ferulic acid and chlorogenic acid, respectively. The only active ingredient that was not identified in any of the analyzed extracts is quercetin.

### 3.2. Determination of MICs of MOEs

The determination of the MIC value of the plant extracts against *Xanthomonas campestris* pv. *campestris* was tested by the microplate assay. From a first analysis of MOEs, the infusion and the aqueous extract with maltodextrins were less effective than the hydroalcoholic extracts with maltodextrins and the hydroalcoholic extracts and methanolic without maltodextrins (Table 4). As shown in Table 4 was clear that both the MOE and WMD-MOE did not show significant antimicrobial activity as they were neither bacteriostatic nor bactericidal at the highest concentration tested. On the contrary, the HA-MOE, MeOH-MOE and HAMD-MOE, showed a complete growth inhibition at 0.5 mg/mL, 0.5 mg/mL, 0.1 mg/mL respectively.

Probably the greatest activity of the hydroalcoholic extracts is closely related to the extraction method, as the use of solvents such as ethanol or methanol allows, according to the principle of molecular affinity, to obtain more concentrated extracts in terms of bioactive molecules constituting the phytocomplex [28]. The results are also showing how the hydroalcoholic extract with maltodextrin (MIC = 0.1 mg/mL) exhibits better antibacterial activity than the hydroalcoholic extract itself (MIC = 0.5 mg/mL).

### 3.3. In Vitro Assessment of Membrane Permeability Alteration

Based on the results, it was assessed whether the extracts with proven antibacterial activity, such as hydroalcoholic extract, hydroalcoholic extract with maltodextrin, and methanol act by changing the permeability of the bacterial membrane.

**Figure 1:**
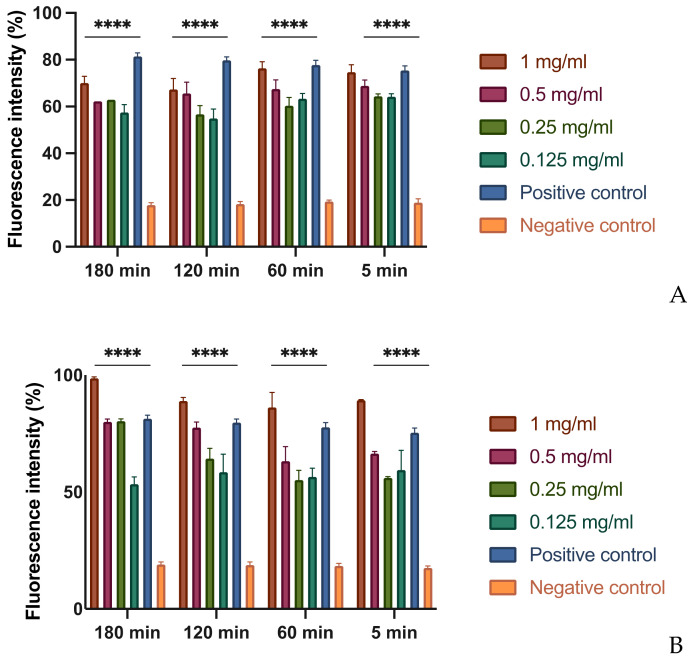

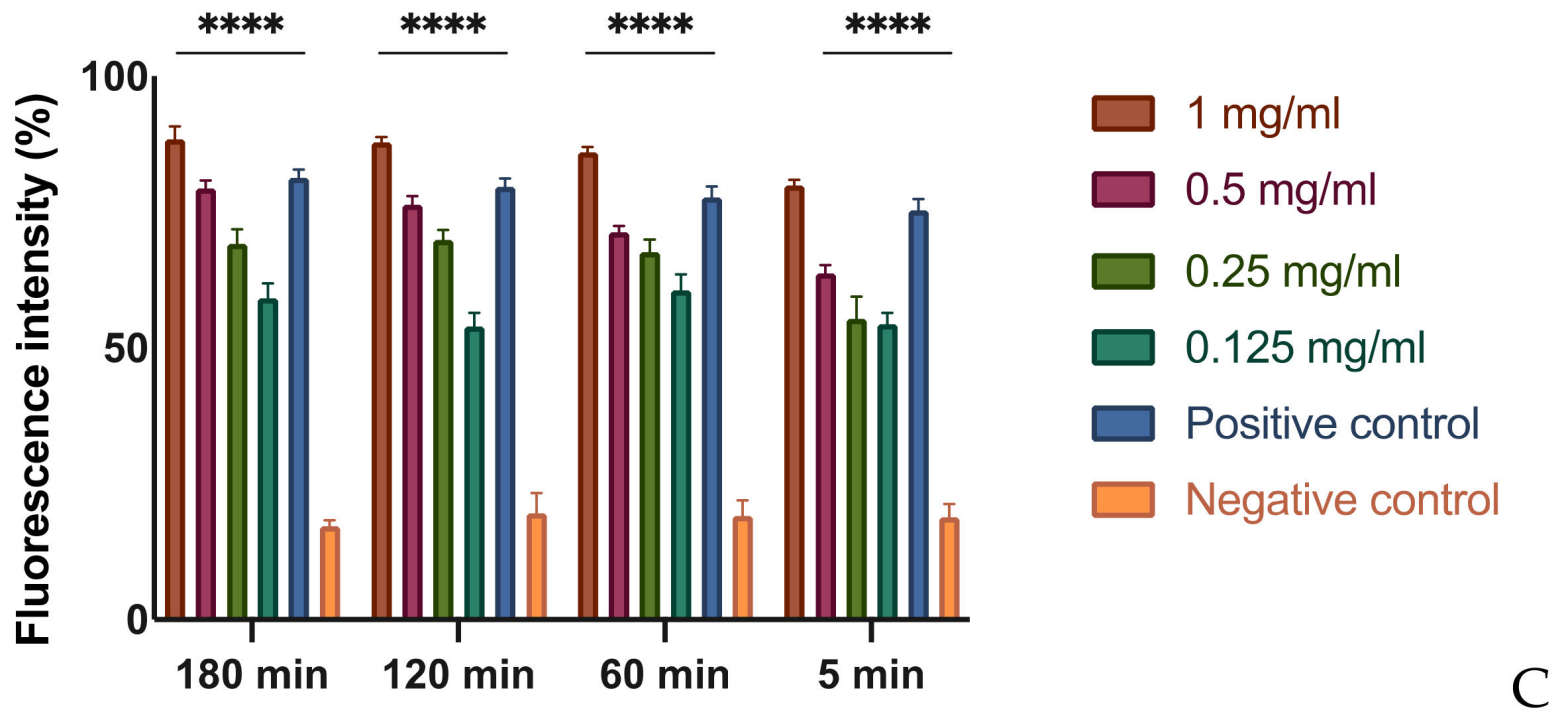
Effects on membrane permeability: (**A**) HA-MOE. (**B**) MeOH-MOE; (**C**) HAMD-MOE. The graph shows the loss of membrane integrity by the increase of fluorescence intensity in the treated samples. Data are the mean of 3 independent experiments performed on triplicate (mean +/− standard deviation), and values are represented as a percentage; **** *p*-values < 0.001.

In order to evaluate this effect, different concentrations of each extract were tested on bacterial suspensions at different times. To demonstrate the possible alteration of the membrane, a fluorescent intercalant agent, propidium iodide, was added to the Xcc bacterial suspensions. This molecule is not permeable to a membrane under normal conditions, but, when the integrity and the permeability of that structure changes, the propidium iodide enters the bacterial cell and intercalates between the DNA bases: this bond increases by 20–30 times the quantum yield and by reading the microplate with the spectrofluorometer allows to differentiate the cells with an intact membrane from those with a damaged and dysfunctional structure [29]. As expected, at the MIC concentration of MOEs, there is an alteration of the permeability equivalent to the positive control represented by the bacteria treated with bleach (Figure 1). It is important to note that even at lower concentrations, such as 1/2 and 1/4 of the MIC, the permeability of the bacterial membrane to propidium iodide increases, although less significantly. This result indicates that, even at low concentrations and already after 5 min of contact, extracts obtained with alcoholic solvents undermine the integrity and permeability of the bacterial membrane.

### 3.4. In Vitro Assessment of Bacterial Swarming

Among the virulence factors of Xcc is extremely important, especially in the phase of invasion of the plant vascular system, the activity of the external aages, such as pili and flagella. To verify if MOEs were capable to inhibit bacterial swarming, petri dishes were prepared by mixing soft LB agar (0.4% agarized soil), nitro blue tetrazolium chloride as a dye and each MOE, at three different concentrations lower than the MIC. Then, a bacterial suspension was inoculated into the center of the petri dish. The soft agar allowed the swarming of *Xanthomonas campestris* pv. *campestris*, and the movement was detected thanks to the activity of nitro blue tetrazolium chloride: this compound, is able to interact with the enzyme NADPH-oxidase, an enzyme capable of transferring electrons alternately to O_2_, with H_2_O_2_ formation, or to salt, with formation of an insoluble blue-black precipitate (formazan) [30].

**Figure 2:**
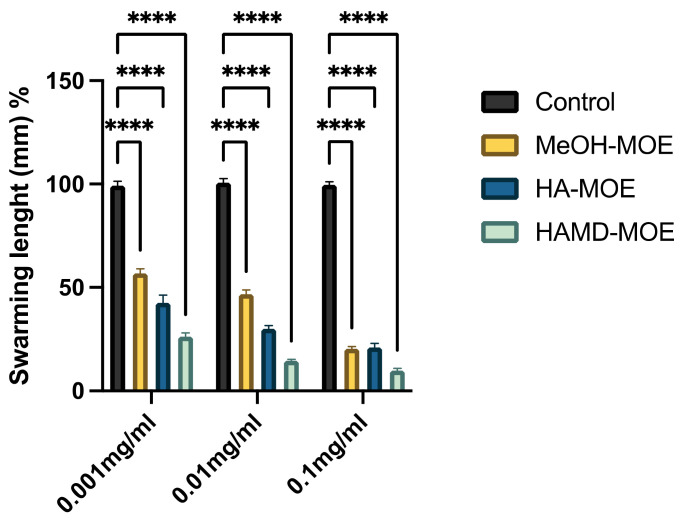
Effects of MOEs on swarming motility. The Xcc movement is measured in mm; the measurement of the swarming area was taken from the point of inoculation and presented as the percentage compared to the control. Data are the mean of 3 independent experiments performed on triplicate (mean +/− standard deviation), and values are represented as a percentage; **** *p*-values < 0.001.

**Figure 3:**
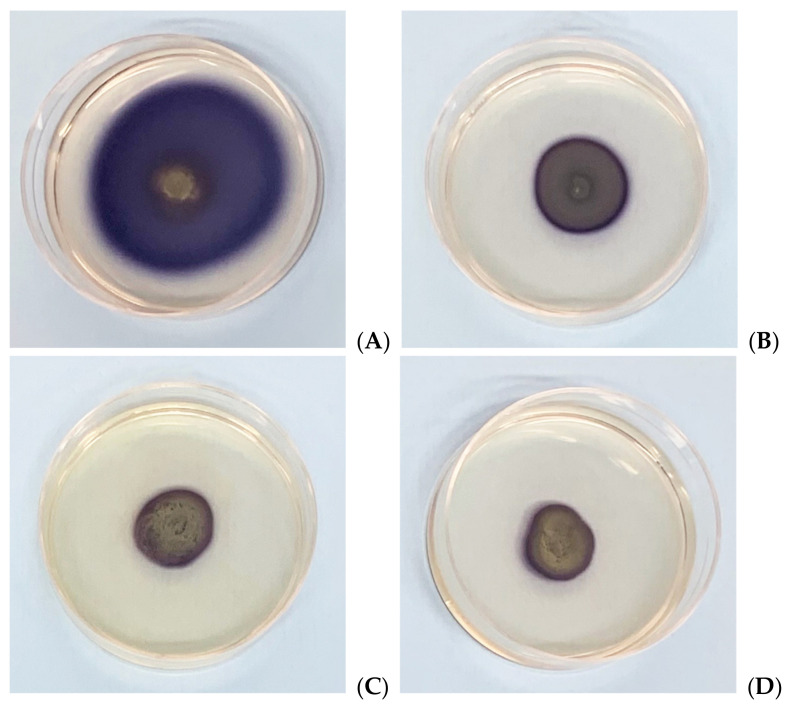
Effects of MOEs on swarming motility; images represent soft-agar plates inoculated with Xcc; the colored area is formed by formazan metabolized by the swarmed bacteria; (**A**) control, (**B**) HAMOE 1 mg/mL; (**C**) MeOH-MOE 1 mg/mL; (**D**) HAMD-MOE 1 mg/mL.

The results show that for each tested extract at different concentrations, both the length and the displacement area of movement decrease significantly with respect to the control, consisting of untreated bacteria (Figure 2 and Figure 3). Presumably, this effect could be due to a depletion of ATP in the bacterial cell when it meets the phytocomplex present in the MOEs. In this sense, molecules that interfere with the cytoplasmic membrane interrupt the driving flow-force generated by protons and electrons, involving a dissipation of the membrane potential and an alteration of the electrochemical gradient, essential elements for the synthesis of ATP needed for movement and chemotaxis [31].

### 3.5. In Vitro Assessment of Biofilm Formation

**Figure 4:**
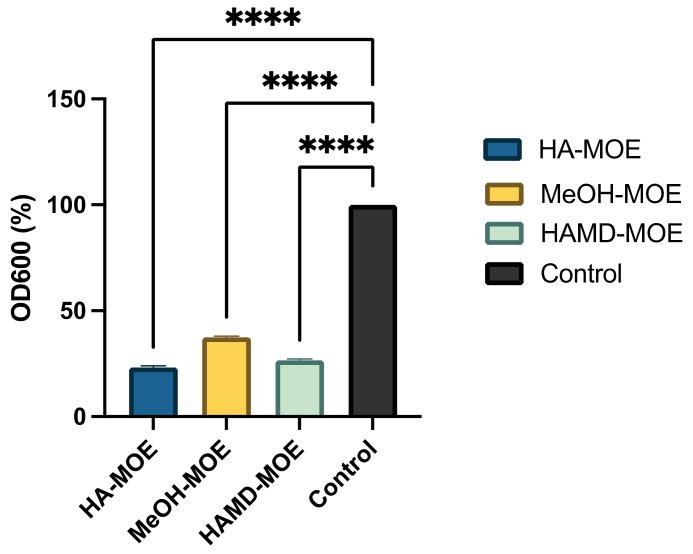
Effects of MOEs on biofilm formation: The Xcc biofilm was measured by OD_600_ and presented as the percentage compared to the control. Data are the mean of 3 independent experiments performed on triplicate (mean +/- standard deviation), and values are represented as a percentage; **** *p*-values < 0.001.

Biofilm formation is an essential mechanism in the pathogenesis of black rot on crucifers. It is a phenomenon that allows the bacterial colony to exercise resistance in a homogeneous and cohesive way, thanks to the various intercellular communication mechanisms enhanced under stressful environmental conditions. To assess whether MOEs had anti-biofilm activity, concentrations below MIC of MOEs were added to the bacterial suspension. After incubation, biofilm formation was quantified by reading the plate with a spectrophotometer. In Figure 4, if compared to the positive control, consisting only of the bacterial suspension, with all MOEs tested, it is notable a clear decrease in the formation of the biofilm. In this case, the HA-MOE seems to be the most effective, reducing biofilm formation by 77%, compared to the control. Additionally, the HAMD-MOE and MeOH-MOE shows a reduced biofilm formation by 73% and 62% respectively. These results confirm the hypothesis that some phenolic compounds, such as quercetin, rutin, ellagic acid and others can act as anti-biofilm compounds [32].

### 3.6. Antimicrobial Effects on Radishes

To define the capacity of the HA-MOE, HAMD-MOE and MeOH-MOE to act against Xcc, a preliminary study was conducted to investigate the antibacterial effect on a plant system. Such an experiment can give us indications on how the bacterium interacts with plant cells and on the effect of MOEs in a more realistic, three-dimensional context. The infected plant, in fact, reacts to the attack of the microorganisms activating defensive mechanisms, innate or acquired, which come into play at different levels.

**Figure 5:**
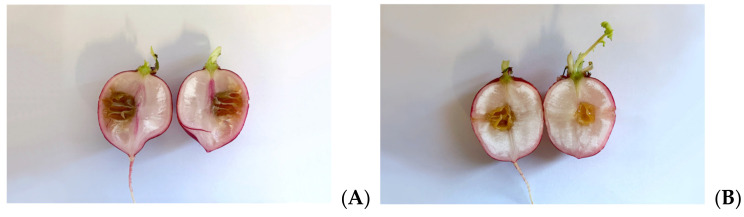

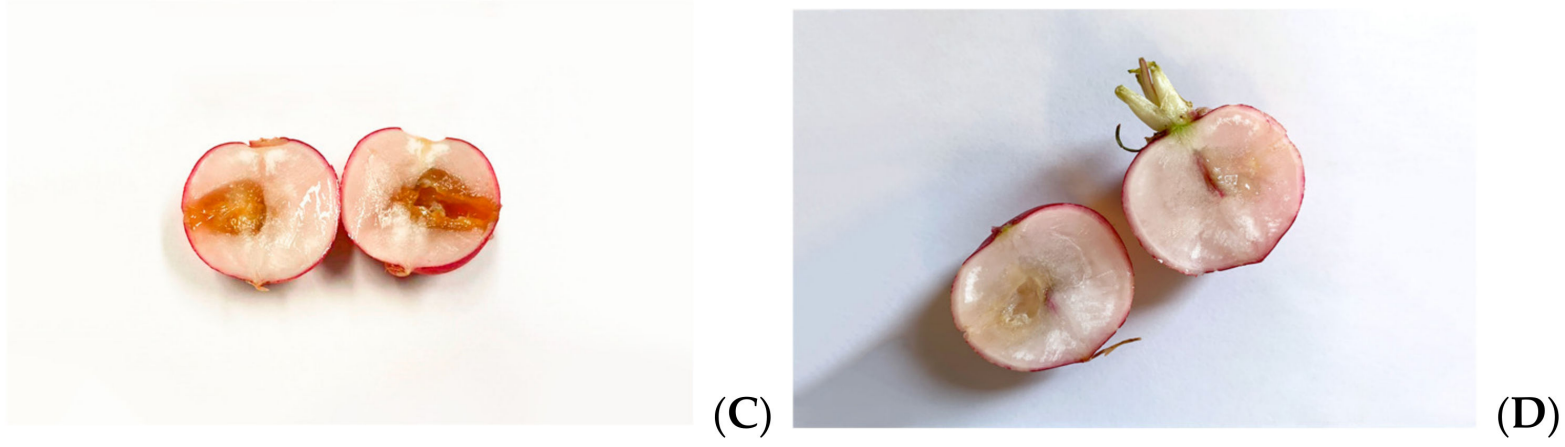
Effects of MOEs in infected radishes: (**A**) control, (**B**) HA-MOE, (**C**) HAMD-MOE, (**D**) MeOH-MOE.

**Figure 6:**
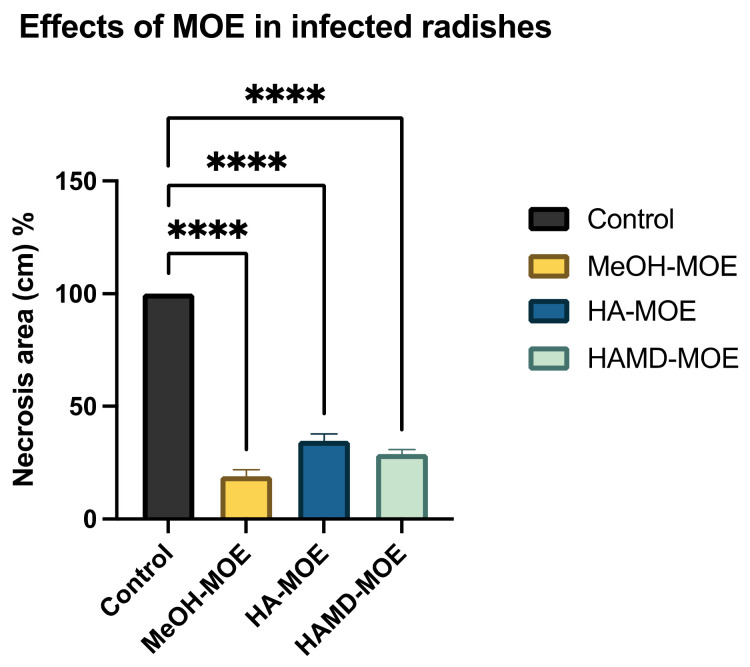
Effects of MOEs in infected radishes. The Xcc infection in radishes was measured by ImageJ quantification of the necrotic area and presented as the percentage compared to the control. Data are the mean of 3 independent experiments performed on triplicate (mean +/− standard deviation), and values are represented as a percentage; **** *p*-values < 0.001.

As Xcc is a causative agent of black rot in crucifers, we tested the antimicrobial properties of such extracts on radishes, previously infected with the bacterium. At the end of the incubation period, radishes were cut to measure the possible area of infection. As can be seen in Figure 5, although all the tested extracts reduce the area of infection with significance, no one was able to eradicate it completely, as necrosis area were still present after the treatment. Anyhow, the methanol extract seems to be the most effective, reducing by 80% the necrosis area compared to the control; also, HA-MOE and HAMD-MOE had shown a reduced infected area, by 65% and 71% respectively (Figure 6). These results can give us important indications on what are the possible candidate molecules that exert this antibacterial effect but, also in view of a possible application in the agricultural field, this affirmation requires further analysis.

## 4. Discussion

An initial analysis of the minimum concentration was carried out, showing a bacteriostatic and bactericidal effect of MOEs. The results obtained show that the infusion and the aqueous extract with maltodextrins do not show antibacterial activity at the concentrations tested while the hydroalcoholic extract, the hydroalcoholic with maltodextrin and methanolic extracts showed bacteriostatic and bactericidal effect at concentrations of 0.5 mg/mL, 0.1 mg/mL and 0.5 mg/mL, respectively. Probably, the greatest effect is due to the extraction method such that the use of solvents of an alcoholic nature, for the principle of molecular affinity, allows to obtain extracts much more concentrated in terms of bioactive molecules. As was shown in Table 3, the most present active ingredients were found to be ferulic acid, rutin and chlorogenic acid. HA-MOE was in fact the extract that showed the highest concentration of polyphenols, presenting an excellent percentage of rutin and ferulic acid, and great results in all performed experiments. HA-MOE, HAMD-MOE and MeOH-MOE are comparable in terms of activity and in terms of polyphenol percentage, but the highest effect of the maltodextrin hydroalcoholic extract is probably due to the presence of maltodextrins which are generally added as processing aids in spray drying processes, as they act as coating agents incorporating bioactive molecules, prolonging their shelf life and preventing their loss of activity [33]. In fact, a study conducted in 2019 by Sri Harsha et al. observed that the presence of maltodextrins significantly improves the stability of certain polyphenolic compounds, in particular chlorogenic acid [34].

### 4.1. Structure-Activity Considerations

**Figure 7:**
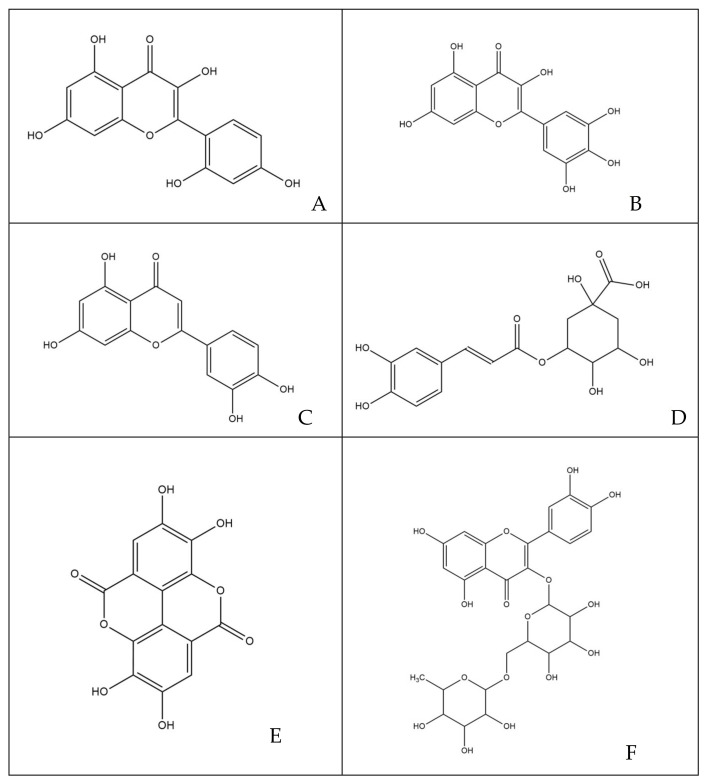

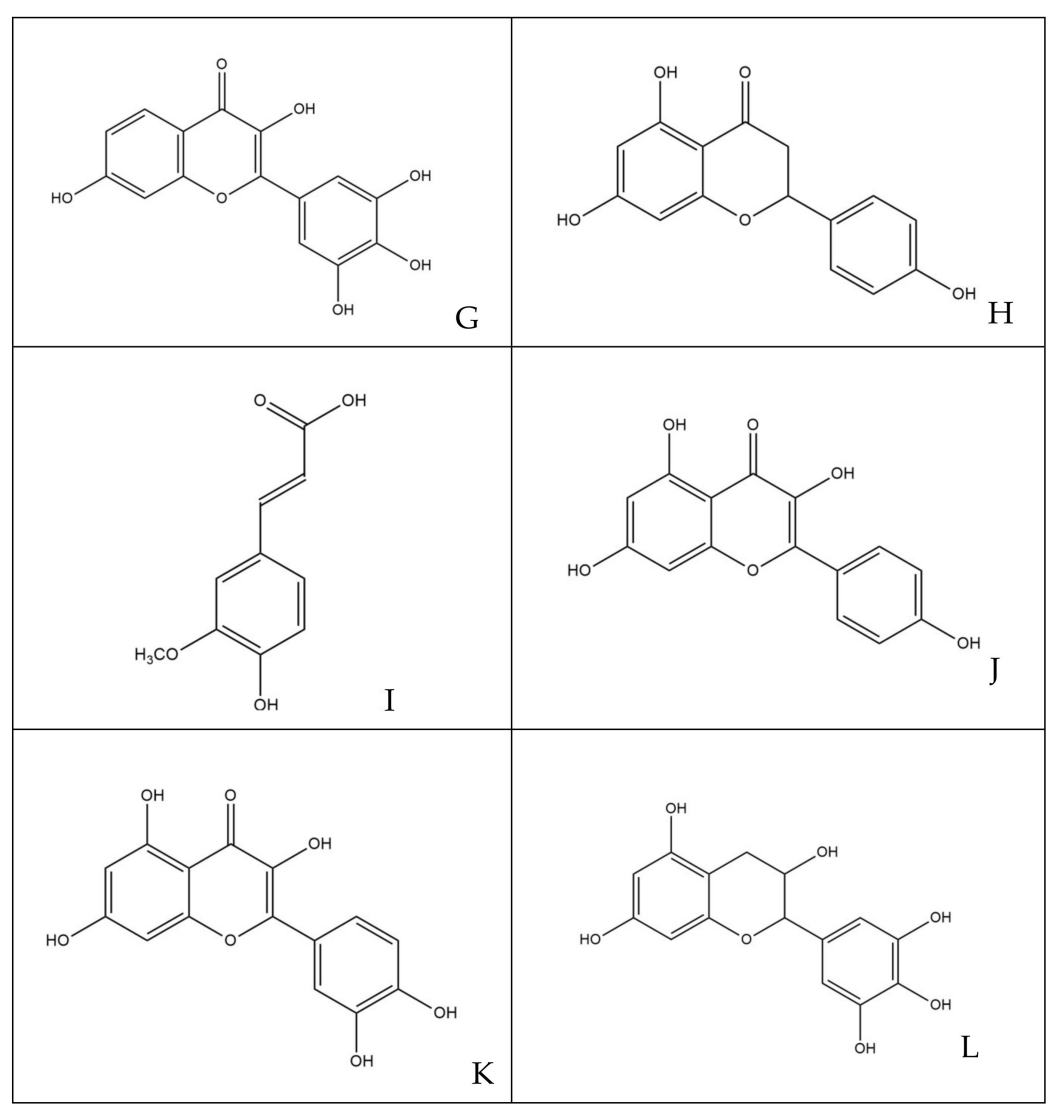
Structures of molecules typically found in MOE phytocomplex [14,15]: (**A**) morin; (**B**) myricetin; (**C**) luteoline; (**D**) chlorogenic acid; (**E**) ellagic acid; (**F**) rutin; (**G**) robinetin; (**H**) naringenin; (**I**) ferulic acid; (**J**) kaempferol; (**K**) quercetin; (**L**) epigallocatechin.

The phytocomplex mainly consists of polyphenolic molecules able to alter the permeability of bacterial membrane leading to a halt in the synthesis of ATP, resulting in the slowing down of all ATP-dependent functions and reduced selectivity towards compounds that may enter the bacterial cytoplasm [32]. In addition, some molecules act by altering the permeability and integrity of the membrane by favoring the passage in the cytoplasm of compounds that are able to inhibit enzyme complexes involved in bacterial replication [35]. Specifically, among the various simple phenols, between the flavonoids and phenolic acids detected in the leaves of *M. oleifera* Lam., only rutin, naringenin, chlorogenic acid, ferulic acid and ellagic acid can complex to bacterial cell walls, altering the fluidity, the structure and functionality of the double phospholipidic layer [36]. Some molecules, such as robinetin, myricetine and epigallocatechin, once they overcome the bacterial membrane, seem to act by forming hydrogen bonds with the nucleic bases, inhibiting the synthesis of DNA and RNA [37]. Flavonoids with hydroxyl central ring, such as, for example, quercetin and kaempferol, bind to the β-subunit of DNA gyrase, blocking the binding site for ATP and, therefore, compromising processes such as cell division and chromosomal replication resulting in inhibition of bacterial growth. Other flavonoids, such as morin, myricetine and luteoline, seem to inhibit the activity of the enzyme elicase, responsible for the spatial organization of DNA filaments in the bacterium [38]. In addition, the glycosylate flavonol rutin is able to inhibit the type II topoisomerase enzyme, promoting DNA cleavage and, while myricetine seems to inhibit several enzymes of fundamental importance such as dihydrofolate-reductase and different DNA- and RNA-polymerases [32]. All cited molecules are reported in Figure 7.

The interactions between these molecules and membrane phospholipids and proteins therefore lead to a distortion of its physical structure, altering its integrity and functionality. The analysis on the alteration of the permeability of the bacterial membrane has, in fact, demonstrated why all the hydroalcoholic and methanolic extracts of *M. oleifera* have been able, at different times and concentrations, to modify this structure. The mechanism of action by which such molecules interact with bacterial membrane, altering its structure and functionality, is not yet fully understood, however some studies hypothesize a structure-activity relationship in which such molecules with highly lipophilic portions interact with lipids of the phospholipid layer and molecules with high oxidizing-reductive potential interact with membrane proteins, resulting in a distortion of lipid-protein interaction [36]. In detail, phenolic compounds poor in hydroxyl groups and rich, instead, of alkyl chains, by their lipophilic nature seem to interact with membrane lipids, dissolving in the double phospholipidic layer, presumably aligning between fatty acids chains. This distortion of the physical structure would cause expansion, with formation of channels, and destabilization of the membrane which, in turn, would increase its degree of permeability [39]. Hydroxyphenols, flavonols and quinones, molecules characterized both by lipophilic portions, able to interact with the membrane, and by a high redox potential, seem to complex irreversibly with the nucleophilic amino acids of membrane proteins, causing their denaturation [35]. Additionally, essential oils play a role in the membrane alteration: Marruffo et al. highlighted that essential oils found in *M. oleifera* Lam. leaves (such as hexacosane, pentacosane and heptacosane); their hydrophobicity represents the principal responsible for the distribution in bacterial structures, but mainly in Gram-positive bacteria, altering all the processes that take place in the bacterial membrane (membrane-coupled energy-transduction processes, solute transport, metabolism regulation and turgor pressure control) [40]. Additionally, hydrolysable tannins, such as ellagic acid, seem to complex with the membrane and to bind proteins nonspecifically through hydrogen bonds, covalent bond and hydrophobic interactions, causing the deactivation of microbial adhesines and membrane transport systems [41]. In addition, some phenolic molecules such as, for example, catechins, rutin, quercetin and kaempferol appear to inhibit various enzymes involved in the biosynthesis of structural elements, especially fatty acids of the bacterial membrane and peptidoglycan, such as the enzyme FAS II (fatty acid syntase II), the transacyclases protein regulating the enzyme FAS II, 3-ketoacyl-ACP reductase and enoyl-ACP reductase, thus blocking the repair of any damage to the membrane [32,42].

The modification of membrane integrity and permeability results in a considerable dissipation of energy since, in fact, it implies the dissipation of the action-potential and the alteration of the electrochemical gradient, conditions necessary for the synthesis of ATP [31]. This condition has, therefore, consequences on various ATP-dependent mechanisms, such as motility: by the analysis carried out, in fact, treated Xcc, subjected to energy shortages, has a smaller area and a shorter length of movement compared to the control. In addition, the swarming of the bacterium is a process finely regulated by a sensor system that perceives and records the characteristics of the surrounding environment, to indicate the direction towards more favorable surroundings, promoting the survival of the bacterium: in this sense, the bacterium will try to limit its movement as much as possible to reduce contact with the extract present [43].

The lower efficiency of all ATP-dependent mechanisms also means slowing down, if not arresting, all processes involved in the secretion of molecules necessary for intercellular communication and, finally, the formation of the biofilm. Such reason, probably, would explain the anti-biofilm effect of the three extracts of *M. oleifera* Lam. analyzed on Xcc isolates. It has been shown, in fact, that several polyphenols such as phenolic acids, flavonoids, hydrolyzable tannins and catechins inhibit the formation of biofilms by influencing ATP-dependent mechanisms of bacterial regulation such as quorum-sensing or other global regulatory systems: the ability of some catechins and flavonols to effectively counteract cell adhesion, the initial stage of biofilm formation, was found in *E. coli* and *S. aureus* [44]. Ellagic acid, tannic acid and epigallocatechin-gallate, on the other hand, seem to inhibit the maturation of the biofilm, an effect probably related to wall damage due to the cleavage of peptidoglycan [45]. As the anti-biofilm activity of the extracts has been defined, more in-depth studies will be necessary to evaluate the stage of formation of the biofilm that is most compromised.

### 4.2. Effects on Infected Plants

Once outlined, the in vitro activity of the hydroalcoholic, hydroalcoholic with maltodextrin and methanol extracts on Xcc, was subsequently evaluated the effectiveness of the MOEs on an infected plant system, such as the root of *Raphanus sativus*. Infection by Xcc in the fleshy root of radish leads to maceration of plant tissue with the formation of cavities occupied by bacterial slime. Radish root is, in fact, an organ rich in water and nutrients, especially sugars, which make it the ideal environment for colonization by the bacterium. The inoculation of MOEs, although, resulted in just a minimal decrease in the area of infection: presumably, the antimicrobial concentration of each extract found in vitro, following inoculation, dilutes into the water-rich root, decreasing the effect found in our previous experiments. This may also be due to the fact that the tested concentrations of the extracts may not be able to inhibit the Type III Secretion System (T3SS), one of Xcc characteristic virulence factors that is activated, solely and exclusively, when the bacterium comes into contact with the target cell, via the interaction between a specific prokaryotic cell protein and a receptor present on the plant cell [46]. This characteristic explains why its possible inhibition cannot be documented by in vitro experiments. Despite, therefore, an effective inhibitory action of T3SS by various phenolic molecules has been documented, the tested concentrations of *M. oleifera* Lam. extracts have not led to a satisfactory result [46]. Consequently, although the results do not appear to be consistent with what was previously determined, further analysis will be necessary to confirm the result. In particular, further experiments are planned in order to test increasing concentrations of MOEs and, if effective, perform an analysis *in planta*, verifying the defensive response settled up by the plant as a whole system.

## 5. Conclusions

The disease of black rot has a great impact in agriculture, especially from an economic point of view, due to the lack of effective bactericides, also for the environmental impact of the use of aggressive pesticides and for the development of resistance resulting from the massive use of non-specific antibiotics. The aim to reduce the use of toxic products to avoid adverse effects on human health and on the environment, as well as the appearance of resistant bacteria, has prompted research to investigate more sustainable alternatives. Among the alternatives, the use of phytocomplexes with antimicrobial properties seems to be an effective and eco-sustainable strategy. From our studies, therefore, the antibacterial activity of the extracts of leaves of *M. oleifera* Lam. on the bacterium *Xanthomonas campestris* pv. *campestris* looks promising: the multiple and synergic mechanisms of action involved with the phytocomplex, the high efficacy, eco-compatibility and sustainability that it would imply, low cost and low risks, make these extracts ideal as potential means of controlling black rot in crucifers.

## Figures and Tables

**Table 1:** List of Xcc bacterial isolates.

BACTERIA	Sample ID	Host	Province of Isolation	Year of Isolation
*Xanthomonas campestris* pv. *campestris*	10863	BRASSICA seeds	Bologna	2011
*Xanthomonas campestris* pv. *campestris*	11043	BRASSICA seeds	Bologna	2011
*Xanthomonas campestris* pv. *campestris*	15616	BRASSICA seeds	Forlì-Cesena	2011
*Xanthomonas campestris* pv. *campestris*	15619	BRASSICA seeds	Forlì-Cesena	2012
*Xanthomonas campestris* pv. *campestris*	15622	BRASSICA seeds	Forlì-Cesena	2012
*Xanthomonas campestris* pv. *campestris*	30788	BRASSICA seeds	Ravenna	2014
*Xanthomonas campestris* pv. *campestris*	3586	BRASSICA	DSMZ	1995

**Table 2:** Total phenol and flavonoid content of leaf extracts of *M. oleifera* Lam.

	Total Phenol Content (µg GAE/mg)	Total Flavonoid Content (µg QE/mg)
HA-MOE	45.63 ± 3.41	601.25 ± 44.00
MeOH-MOE	36.37 ± 2.33	1611.98 ± 70.44
In-MOE	37.56 ± 2.77	193.88 ± 1.74
WMD-MOE	25.65 ± 1.20	176.67 ± 9.56
HAMD-MOE	42.49 ± 1.39	291.07 ± 14.91

**Table 3:** Percentages of chlorogenic acid, rutin, ellagic acid, ferulic acid, and quercetin in the five different dried extracts of *M. oleifera* Lam. leaves. Each value was obtained from three analyses (mean ± SD).

	Percentage of Detected Compounds (*w*/*w*)				
	Chlorogenic Acid	Rutin	Ellagic Acid	Ferulic Acid	Quercetin
HA-MOE	0.50 ± 0.04	1.05 ± 0.04	0.20 ± 0.01	2.22 ± 0.03	-^1^
MeOH-MOE	0.34 ± 0.02	0.84 ± 0.04	0.20 ± 0.01	1.12 ± 0.11	-^1^
In-MOE	0.29 ± 0.03	0.49 ± 0.01	0.06 ± 0.00	1.17 ± 0.03	-^1^
WMD-MOE	0.43 ± 0.02	0.30 ± 0.02	0.10 ± 0.00	0.35 ± 0.01	-^1^
HAMD-MOE	0.90 ± 0.04	0.63 ± 0.034	0.21 ± 0.01	0.91 ± 0.00	-^1^

^1^ Not detected.

**Table 4:** MIC values: Each value was obtained from three different experiments performed on triplicate (mean +/− standard deviation). No significant difference was observed for each Xcc strain, reason why the results are presented with one value for all strains.

*M. oleifera* Lam. Leaves Extract	MIC (mg/mL)
Infusion (In-MOE)	>2
Hydroalcoholic extract (HA-MOE)	0.5
Methanolic extract (MeOH-MOE)	0.5
Water extract with maltodextrins (WMD-MOE)	>2
Hydroalcoholic extract with maltodextrins (HAMD-MOE)	0.1

## Data Availability

Not applicable.
